# A Passerine Bird's Evolution Corroborates the Geologic History of the Island of New Guinea

**DOI:** 10.1371/journal.pone.0019479

**Published:** 2011-05-06

**Authors:** Kristy Deiner, Alan R. Lemmon, Andrew L. Mack, Robert C. Fleischer, John P. Dumbacher

**Affiliations:** 1 Department of Vertebrate Zoology and Anthropology and Center for Comparative Genomics, California Academy of Sciences, San Francisco, California, United States of America; 2 Genomic Variation Lab, University of California Davis, Davis, California, United States of America; 3 Department of Scientific Computing, Florida State University, Tallahassee, Florida, United States of America; 4 Powdermill Nature Reserve, Carnegie Museum of Natural History, Pittsburgh, Pennsylvania, United States of America; 5 Center for Conservation and Evolutionary Genetics, National Zoological Park, Smithsonian Institution, Washington, D.C., United States of America; University of Veterinary Medicine Hanover, Germany

## Abstract

New Guinea is a biologically diverse island, with a unique geologic history and topography that has likely played a role in the evolution of species. Few island-wide studies, however, have examined the phylogeographic history of lowland species. The objective of this study was to examine patterns of phylogeographic variation of a common and widespread New Guinean bird species (*Colluricincla megarhyncha*). Specifically, we test the mechanisms hypothesized to cause geographic and genetic variation (e.g., vicariance, isolation by distance and founder-effect with dispersal). To accomplish this, we surveyed three regions of the mitochondrial genome and a nuclear intron and assessed differences among 23 of the 30 described subspecies from throughout their range. We found support for eight highly divergent lineages within *C. megarhyncha*. Genetic lineages were found within continuous lowland habitat or on smaller islands, but all individuals within clades were not necessarily structured by predicted biogeographic barriers. There was some evidence of isolation by distance and potential founder-effects. Mitochondrial DNA sequence divergence among lineages was at a level often observed among different species or even genera of birds (5–11%), suggesting lineages within regions have been isolated for long periods of time. When topographical barriers were associated with divergence patterns, the estimated divergence date for the clade coincided with the estimated time of barrier formation. We also found that dispersal distance and range size are positively correlated across lineages. Evidence from this research suggests that different phylogeographic mechanisms concurrently structure lineages of *C. megarhyncha* and are not mutually exclusive. These lineages are a result of evolutionary forces acting at different temporal and spatial scales concordant with New Guinea's geological history.

## Introduction

**Figure 1:**
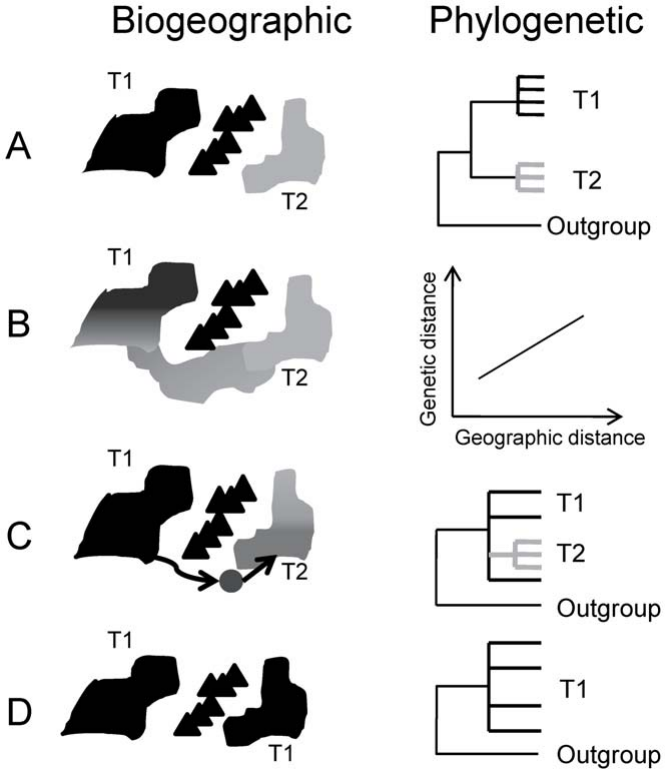
Biogeographic and phylogenetic hypothesis for different evolutionary mechanisms. Biogeographic hypotheses and predicted phylogenetic gene tree patterns are depicted and highlight three evolutionary mechanisms. T1 and T2 represent different taxa. a) Vicariant event where all gene trees within a taxon should be concordant. Note that there may be a required amount of time (expected to be about 4N_e_ generations) before lineage sorting is complete and groups are reciprocally monophyletic at a majority of loci and this visualization does not include complications such as incomplete lineage sorting. b) Isolation by distance with a positive relationship between geographic distance and genetic distance, no gene tree pattern predicted. c) Founder effect with dispersal (black arrows), gene trees are predicted to have a nested, comb-like phylogeny and be concordant within a taxon. d) Panmixia (null hypothesis) with no genetic differentiation between geographic regions. Multiple mechanisms can produce similar genetic patterns; therefore, patterns in tree topology to infer a biogeography mechanism should not be used to the exclusion of other independent lines of evidence.

Phylogeography integrates geographical information and DNA-based genealogies to infer the evolutionary history of modern taxa [1]. Specific patterns in geography and genealogy provide evidence of processes driving lineage divergence often resulting in speciation. In particular, genetic variation becomes correlated with geography via three major hypothesized evolutionary mechanisms: 1) allopatric divergence (AD; such as that caused by vicariance events), 2) isolation by distance (IBD), and 3) founder effect with dispersal (FED) [1], [2], [3], [4], [5], [6]. Each mechanism produces a distinct genetic pattern that can be used to test biogeographic hypotheses (Figure 1). Our study focuses on the island of New Guinea, which has influenced many ideas in biogeography such as Wallace's Line [7] and Mayr's biological species concepts [8], [9]

New Guinea is the world's largest tropical island, roughly 786,000 km^2^, located north of Australia, from 12°S latitude to the equator. It is home to an unusually diverse biota, supporting over 8.6% of the world's bird species in under 0.75% of the world's land area [10]. New Guinea has approximately the same number of bird species as the Australian continent that is nine times larger [11], [12]. New Guinea lowlands currently have few apparent geographic isolating barriers. Even though high mountains separate the north and south coasts, lowland forests form a continuous ring around the island and many lowland species are widely distributed in a ring-like fashion around the island.

Despite having broad geographical ranges in continuous habitat, many lowland animal species have diversified tremendously. For example, birds are among the best-studied animals in New Guinea, and there are many taxonomic breaks at the species and subspecies level across the island [10]. Surprisingly, many of these breaks between groups occur in the same geographic locations, some with few or no obvious current barriers to gene flow and have remained untested with genetic information [10]. The geological literature, however, describes several barriers in the late Pliocene and early Pleistocene that were potentially concurrent with avian diversification, see Table 1 [13], [14], [15], [16], [17], [18], [19].

**Table 1:** Summary of lowland New Guinea geological events relevant to this study.

**Name of lowland region**	**Accretion or formation date**
Bintuni Basin (BB)	3–5 Mya or older; separate island connection date unknown
Meervlakte Basin (MB)	1.5–2.5 Mya; after Central Ranges were formed
Sepik and Ramu Basin (SRB)	1.5–2.5 Mya; after Central Ranges were formed
Cape Vogel Basin (CVB)	Predates 5 Mya, probably not land bridged to New Guinea until 5 Mya
South Papuan Peninsula (SPP)	Predates 5 Mya, probably not land bridged to New Guinea until 5 Mya
South Papuan Basin (SPB)	Lowlands present, but very narrow against steep mountains at 5 Mya; gradually filling with sediment until present extent is reached
Trans-Fly Lowlands (TF)	500 Kyr; land bridged to Australia during late Pleistocene glaciations
**Dispersal Barrier**	**Time Feature Present**
Central Ranges - cr - (uplift)	4–5 Mya (split northern and southern watersheds)
Border Mountains - bm	Mountainous island arcs; predate the formation of northern basins
Huon Peninsula - hp - (uplift)	Mountain uplift, high mountains by 1.5 Mya
Milne Bay – mb	Eastern extent of Central Ranges; probably predates 5 Mya
Aure Trough - at	Inland sea filled by 3–4 Mya
**Dispersal Route**	**Time Route Opened**
Aetna Bay - ab	Connection dates not known
Aure Trough filled - at	Connected SPP to SPB by 3–4 Mya; dates disputed
Trans-Fly Lowlands-tf	Connected TF to Australia as recently as 21 Kyr

The focal species for this study, *Colluricincla megarhyncha* (Little Shrike-thrush, Family Colluricinclidae), exhibits a tremendous amount of morphological variation across its range with thirty recognized subspecies [20], [21]. Thirteen of these subspecies occur on mainland New Guinea, nine occur on small islands off the continental shelf of New Guinea, seven occur in Australia, and one occurs on Sanga Island off of Sulawesi. *C. megarhyncha* is a small (35 g mean weight) passerine that feeds primarily on understory arthropods in or near the rainforest interior [22], [23]. It has habitat associations with tropical gallery, monsoon, rainforests thickets, and in subtropical and littoral forest [24]. Its elevation range spans from sea level to approximately 1200 m, although it is known to inhabit higher elevations (up to 2800 m) in a few localities [20], [21]. Its vocalizations are loud and characteristic, and it responds to conspecific calls, making them easy to locate and observe. Of 545 band recoveries (from 1481 captures), the maximum documented movement was 3 km (Australian Bird and Bat Banding Scheme, online database). Additionally, a study in the South Papua Peninsula region banded 198 birds and had 72 recaptures where only one recapture was more than 400 m from the original capture site [25] and Mack, unpublished data. *C. megarhyncha* is therefore considered resident and relatively sedentary [24] and citations therein. The large amount of morphological variation and probable small natal dispersal distance suggest there may be considerable genetic variation across the species' range. Thus, it is a good species for examining New Guinea lowland biogeography and for testing hypotheses of evolutionary mechanisms across continuous lowland forests.

Several major geological events in New Guinea occurred within the last 6 million years (Mya; Table 1). We postulated these to be important for generating patterns of genetic variation in *C. megarhyncha*. This upper bound is corroborated by evidence from phylogenetic information between a sister taxon, the White-bellied Pitohui (*Pitohui incertus*) and *C. megarhyncha*, and suggests coalescent times within *C. megarhyncha* are 6 Mya or less [26]. In the Miocene (5–23 Mya), most of New Guinea was submerged except a few isolated oceanic mountain ranges that were north of Australia [15], [16]. This major orogeny was complete by the early Pleistocene (2 Mya), and by 3.5–4.5 Mya the Central Ranges were sufficiently high to begin isolating the northern and southern lowland basins [18]. The majority of today's lowland basins are believed to have formed by the middle Pleistocene. The exact extent of these basins may have changed dramatically during the Pleistocene as sea levels rose and fell. For example, the Trans-Fly and South Papuan basins likely extended southward connecting Australia, New Guinea and the Aru Islands, what is now the Gulf of Carpentaria and the eastern portion of the Arafura Sea [27].

**Figure 2:**
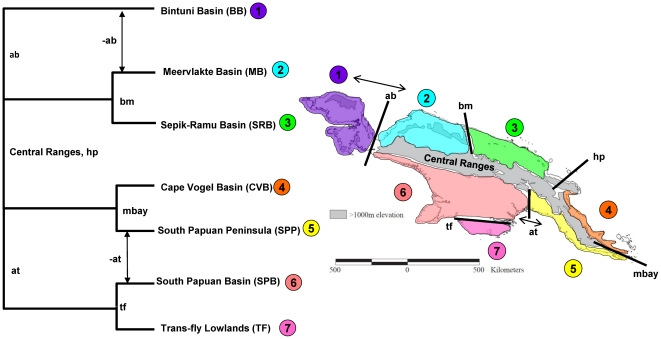
Hypothesis of monophyletic groups in *Colluricincla megarhyncha* for New Guinea lowland basins. (A) Hypothesized cladogram for lowland basins with phylogeographic mechanism of divergence or similarity indicated. Abbreviations near nodes are in Table 1 and are the barriers or potential dispersal routes. (B) Map of vicariance events are noted as follows: The Central Ranges are a barrier for the northern basins and southern basins (grey); The Border Mountains (bm) separating Meervlakte (MB, blue) from Sepik-Ramu (SRB, green); Huon Peninsula (hp) uplift separates SRB from Cape Vogel (CVB, orange); Milne Bay (mbay) filled to Central Ranges creating barrier between CVB and the South Papuan Peninsula (SPP, yellow); Aure Trough (at) separated SPP from South Papuan Basin (SPB, pink); Trans-Fly River separates SPB from Trans-Fly Basin (TF, dark pink) and the Bintuni Basin (BB, purple) was a former island separated from MB and SPB by the Aetna Bay (ab). Hypothesized dispersal events (indicated by arrows) include the Aetna Bay (ab) filled in connecting BB to MB and SPB, the Aure Trough (at) filling in connecting SPP with SPB.

Given the geological evidence (Table 1), we hypothesize the relationship of birds found among basins to have a phylogenetic pattern as depicted in Figure 2, which considers both the vicariant nature of the basins, as well as potential dispersal routes that may currently connect populations. The tree depicts relationships among extant taxa and is not meant to imply a single colonization event. We used DNA sequence variation from three mitochondrial (mtDNA) regions (NADH dehydrogenase 2 [ND2], cytochrome b [CytB], and a region containing tRNA-lysine and ATPase8 genes [ATPase8]) and one nuclear intron (nDNA) region (RNA fingerprint protein 35 [AR35]) to evaluate genetic patterns among sites sampled from putative geographic basins. Genetic diversity among geographic locations was then used to test what potential role the mechanisms of allopatric differentiation, isolation by distance, and founder effect with dispersal have had in structuring *C. megarhyncha* populations throughout New Guinea and surrounding islands.

## Methods

### Ethics Statement

This study was carried out in strict accordance with the recommendations in the Guide to the Use of Wild Birds in Research (Gaunt and Oring, 1997). Blood samples were taken from the brachial (wing) vein and any birds collected were euthanized using recommended techniques and all efforts were made to minimize suffering. Field protocols were approved by the Smithsonian National Zoological Park Institutional Animal Care and Use Committee (IACUC). All specimens were exported legally under Papua New Guinea export permits [permit numbers 200263 (2000), 020170 (2002), 020480 (2002), and 990333 (2005)] and imported under USDA Animal and Plant Inspection Services (APHIS) import permits 42797 (to Robert Fleischer) or 50431 (to John Dumbacher) and declared to US Fish and Wildlife Service using form 3–177.

### Sampling

**Figure 3:**
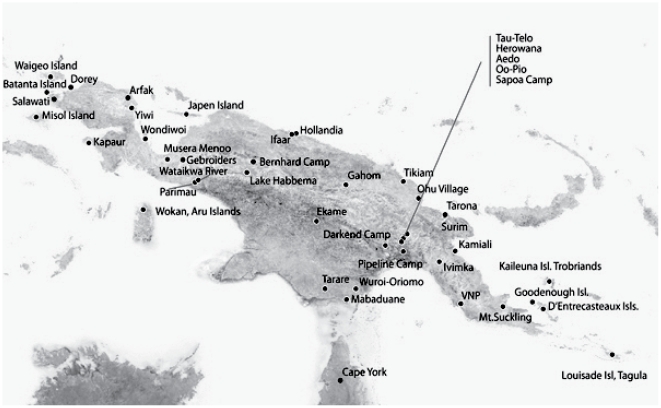
Distribution of sampling localities of *Colluricincla megarhyncha*. Each black dot with corresponding name represents the geographic location from which samples in Table S1 were collected.

We sampled tissues from 174 individuals from 45 localities representing 23 of the 30 described subspecies of *C. megarhyncha* (Figure 3; Table S1), focusing on those that occur on or immediately adjacent to New Guinea. We collected voucher specimens and tissues of *C. megarhyncha* from several basins in Papua New Guinea during expeditions that took place between 2001 and 2006. Additional birds were sampled from museum skins (see Table S1 for description of samples and Table S2 for complete specimen details).

### Molecular protocols

DNA was extracted from fresh tissues using a DNeasy kit (Qiagen), following manufacturer's recommended animal tissue protocol. DNA from museum skins was isolated in a dedicated ancient extraction laboratory in which no modern or post-polymerase chain reaction (PCR) samples are handled. A small toepad fragment was digested overnight at 56°C in lysis buffer (1 mM Tris, EDTA, DTT and proteinase k, pH 8.0), phenol and chloroform extracted, and dialyzed in centrifuge-assisted Centricon® tubes (Amicon, Beverly, MA, USA).

PCR involved a number of different primer pairs [28], [29], [30], [31] to amplify three mtDNA regions and one nuclear intron (see Table 2 for primers and PCR conditions). Because of the degraded nature of DNA isolated from skins, we amplified regions in short fragments of 135–300 bp. Furthermore, every PCR with museum tissues was repeated at least twice for verification purposes for both nuclear and mtDNA loci. PCR involved denaturing at 94°C before thermocycling 35 cycles (profile 94°C denaturing/30 s, variable (see Table 2) annealing temperature/30 s, and 72°C extension/1 min), followed by a 5 minute extension at 72°C. PCR success was assessed by electrophoresis on a 1.2% agarose gel. PCR products were purified and both heavy and light strands were sequenced using dideoxy chain termination chemistry with Big Dye v3.1 following recommended ABI protocols and run on an ABI3100 automated capillary sequencer. Genbank accession numbers are in Table 2.

**Table 2:** Primers, PCR conditions and GenBank accession numbers for loci and sequences used in this study.

Locus	Length	Primer Name	Primer Sequence (5′-3′)	A_t_ (°C)	[MgCl_2_]	Accession numbers	Reference
ATPase 8	195	BirdsRUS (BRUS)	TGGTCGAAGAAGCTTAGGTTC	50	2.5	HM006357-	28
		T-Lys	CACCAGCACTAGCCTTTTAAG			HM006530	29
ND2	242	ND2CM-318F	GATTCCCAGAAGTKCTTCAYGG	50	2.5	HM006531-	This study
		ND2CM-582rc	GTATAGGTAGAARTTGAGTA			HM006701	This study
Cytb	135	CytBwowrc	GACAAAATCCCATTCCACCC	50	2.5	HM006020-	30
		H15573	AATAGGAAGTATCATTCGGGTTTGATG			HM006188	31
AR35	138	AR35-LI3rc	CAGTCCCATGTGGTACCAAG	46	3.0	HM006189-	This study
		AR35-RI1	GCATGCAGTGACACAGACCT			HM006356	This study

Length of region sequenced is in base pairs, At is the annealing temperature used for amplification and magnesium chloride (MgCl2) is in micro molar concentrations.

Sequences were aligned and edited with SEQUENCHER 4.7 (GeneCodes, Ann Arbor, MI, USA). PHASE v2.1 was used to resolve nuclear alleles when heterozygous sequences were detected [32]. Nuclear alleles that were not resolved with greater than a 60% probability were dropped from further analysis. This resulted in 46 out of 336 alleles being dropped from analyses. Many alleles dropped were due to being heterozygotes and the inability to phase the data beyond a 50% probability; none were due to gap variation. We used jModelTest V0.1 [33], [34] to find the best-fit model for our mtDNA data using the Akaike Information Criteria, as well as testing a model that partitioned the data by gene and codon. The general time reversible model with a gamma rate distribution and invariant sites parameter (GTR+I+G) was the best-fit model for these data. Only mitochondrial loci were concatenated and used in subsequent likelihood searches and all analyses. Redundant haplotypes we removed prior to phylogenetic reconstruction. Nuclear alleles were not used in all analyses and we have specified when they were used to estimate a particular statistic.

### Phylogenetic reconstruction with mtDNA only

We searched for the best-fit tree with GARLI v0.951 [35] using the estimated maximum likelihood criterion and a general time reversible model (GTR+I+G). A bootstrap analysis of 1000 replications was performed in GARLI to assess support for nodes. Additionally, we performed a Bayesian phylogenetic analysis using MrBAYES v3.1.1 with default parameters except the following, Lset nst = 6 rates = invgamma; mcmc ngen = 20000000; printfreq = 10000; samplefreq = 1000; nchains = 4 [36]. We used a representative taxon from a closely related species (*Pitohui incertus*) [26] as the outgroup for phylogenetic analysis (Table S1).

### Molecular variance with mtDNA and nDNA

An analysis of molecular variance (AMOVA) was performed with mtDNA pairwise estimates of F_st_ (using the 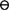
_st_ estimator, but referred to as F_st_ throughout paper) for both mtDNA and nDNA in Arlequin v3.11 [37] to test whether partitioning of genetic variance was significant among the hypothesized putative basins (Table 1, Figure 2). Since classic F_st_ uses haplotype frequencies for mtDNA and there were no instances of shared haplotypes among basins, we estimated F_st_ with the Kimura 2-parameter (K2P) genetic distance, which takes differences among haplotypes into account, as implemented in Arlequin v3.11 [37]. Additionally, a network of haplotypes was constructed separately for both mtDNA and nuclear datasets using median-joining method in NETWORK v4.5.1.2 [38].

### Divergence dating with mtDNA only

Molecular clock estimates of divergence were performed to corroborate the geologic timing of hypothesized isolating barriers (Table 1). We used two methods of dating. First, pairwise K2P genetic distances among putative basins were calculated using Arlequin v3.11 [37] and divergence estimates were divided by an assumed molecular clock. Second, we estimate timing of divergence using BEAST v1.5beta2 [39], [40] which allows for rate variation across the phylogeny.

For the first divergence date estimate, the K2P genetic distance was used because this method takes into account a difference in substitution rates between transitions and transversions while permitting multiple substitutions per site [41], [42]. This is also the closest approximation to the GTR+I+G model used to calculate the genetic distances in our maximum likelihood tree [43]. Consequently, we used the gamma value estimated from the ML search (0.68) to correct the K2P distance as described by Excoffier [37]. A gamma correction is used when it cannot be assumed that the substitution rate is uniform across all sites. We calculated an average between-basin K2P genetic distance for each basin, and corrected it by subtracting the average within-basin distance following the equation (*d_xy_*−(*d_x_*+*d_y_*)/2), where *d_xy_* is the average number of pairwise differences between basins and *d_x_* and *d_y_* are the average number of pairwise differences within each basin being compared [44], [45], [46]. Pairwise molecular divergence levels among basins were converted to dates using Weir and Schluter's [47] estimate of 2.1% per Mya, averaged from 36 independent passeriform calibrations. We acknowledge that there is no available calibrated molecular clock with fossils for closely-related passerines in this geographical region, and we do not mean to imply that this rate is strictly accurate. These are provided to offer some estimate of divergence that can be roughly associated with geographically relevant events in the region.

For the second divergence date estimate we used Bayesian analysis in BEAST v1.5beta2 to estimate dates of various nodes in the reconstructed phylogeny. BEAST allows input of a mean rate as a prior in the analysis, but allows branches to vary in rate locally across the tree [39], [40]. We constructed the parameter and data file in BEAUti v1.5beta2, selecting the general time reversible (GTR) with invariant sites (+I) and gamma distribution of site changes (+G) model of DNA sequence substitution. We used an age-independent model of sequence error, an uncorrelated and relaxed clock model, and Yule species branching model for the tree prior. We incorporated a normally distributed prior rate using the mean estimate and standard error from the meta-analysis of Weir and Schluter [47] as 2.1% (±0.1%). The tree was rooted by establishing a taxon set of the ingroup (i.e., all taxa except the outgroup *Pitohui incertus*). We ran a chain of 20,000,000 generations in BEAST v1.5beta2, logging parameters every 1000 generations. The chain converged by 14,000,000 generations such that effective sample sizes (ESS) for each parameter evaluated in Tracer v1.4.1 were all above 100 and most well above 200 (given a 10% burn-in).

### Population expansion with mtDNA only

Haplotypes within basins were compared to estimate the number of transitions (n_s_), transversions (n_v_), and substitutions (n_d_) as implemented in Arlequin v3.11 [37]. Mean pairwise differences (π) among haplotypes within basins were calculated using Arlequin v3.11 [37] and their corresponding standard deviations are reported. Fu's F [48] and Tajima's D [49] were used to test for patterns consistent with population expansion within putative basins.

### Test of isolation by distance with mtDNA only

Isolation by distance was assessed by testing whether there is a correlation between genetic distances with geographic distances among sampling localities. We calculated geographic distance using Pathmatrix v1.0 [50] which is an extension implemented in the Spatial Analyst for the geographical information system (GIS) software ARCVIEW 3.3 (Environmental Science Research Institute, Redlands, USA). This program calculates geographic distance while taking into account the cost of movement through a landscape. For our species, we know that *C. megarhyncha* is typically restricted to elevations below about 2300 m; therefore we calculated geographic distances between two sites based on the lowest elevation and shortest path. This is called the effective geographic distance and is different from Euclidian distance, which is the shortest path possible, and is typically not realistic for the biology of the species [50]. To estimate the effective geographic distance, a one-meter resolution digital elevation model (DEM) for New Guinea was used to create a “friction grid” composed of weighted cells that impose a cost of moving through that cell based on the average elevation for the cell, and above 2300 m a route was not allowed to be calculated. The cost value of each cell ranged from 1–5096, where an elevation of 1 m above sea level was assigned a cost of 1 and an elevation of 2300 m was assigned a cost of 5096 following the exponential equation y = 1.4142 e ^0.0035x^. This pattern generated a much higher cost for moving between cells at the highest elevations. Cost parameters as they relate to migration distances are not well understood and we chose an exponential increase based on the distribution of the species (e.g. most birds have been sampled at low elevation and very few are found at elevations above 2300 m). Furthermore, there is no difference in the significance of the correlation of distance calculated with a linear or exponential model assumed for the friction grid.

A correlative test for isolation by distance was performed with pairwise K2P genetic distance (the same distance calculated by Arlequin for the F_st_ analysis) and effective geographic distances estimates among sites. Significance of the correlation between genetic distance and effective geographic distance were estimated using a Mantel test in Arlequin v3.11 [37].

### Tests of Evolution of Dispersal Distances with mtDNA only

We used PhyloMapper v1beta1 [51] to test the prediction that dispersal distance has evolved within *Colluricincla*. Following the methods described by Lemmon and Lemmon [51], we used non-parametric rate smoothing to adjust branch lengths in the maximum likelihood tree [52] with the MRCA of the *C. megarhyncha* ingroup set to 5 million years before present. Default settings for the rate smoothing were used for the PhyloMapper analyses. Using the resulting chronogram, we then constrained all branches to a single dispersal class and estimated the mean per generation dispersal distance. A second analysis was then conducted in which branches were divided into five dispersal classes (see Figure 2 for key to abbreviations): 1) the SPB and BB class, 2) the SRB class, 3) the D'Entrecasteaux Island class (Fergusson, Goodenough and Normanby Is.), 4) the SPP class, and 5) the class containing the remaining branches. These five classes were chosen based on each having a sample size of greater than 10 individuals. A final analysis focusing on the SPP clade was conducted in which the branches in the CVB clade were placed in a separate dispersal class from the rest of the branches in the SPP clade. Significance was assessed using two chi-squared tests (one comparing 1 class to 5 classes, and the second comparing 1 class to 2 classes) following methods described in Lemmon and Lemmon [51].

If dispersal distance has evolved in response to changes in range size (e.g. reduced dispersal for island individuals), a positive correlation between the dispersal distances estimated in PhyloMapper analyses and range size estimated by computing the square root of the potential range area within a basin should be observed. To test for this correlation, the area of each geographic basin was estimated using Google Earth Pro (Google, Inc.) and was based on putative barriers outlined in Table 1. Area of range size is coarsely delineated in Figure 2. A correlation test was performed using the correlation test function in R version 2.10.1(using default parameters). No relationship between dispersal distance and range size is predicted under the null hypothesis.

## Results

### Phylogeographic relationships for mtDNA only

**Figure 4:**
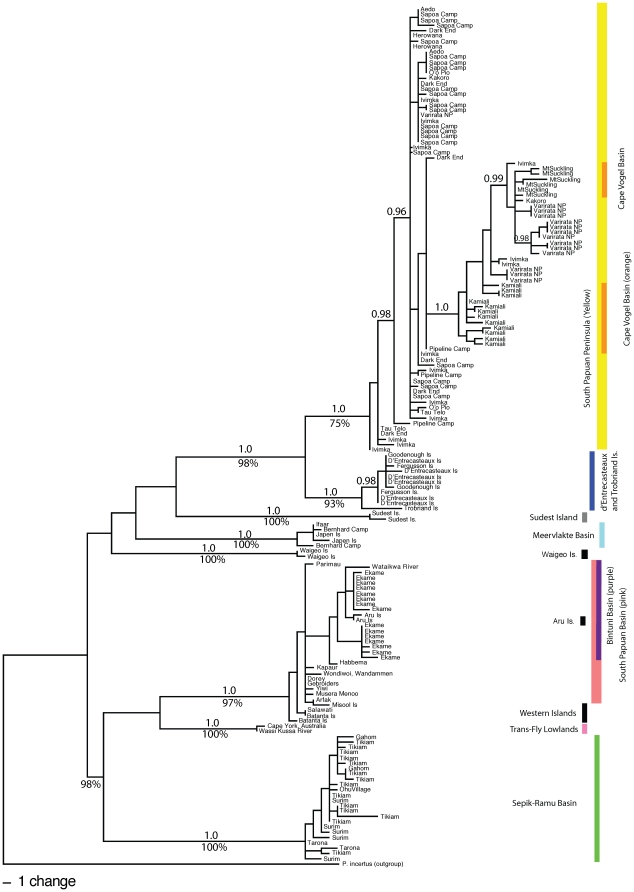
Estimated phylogenetic tree from mitochondrial DNA for 23 of the 30 subspecies of *Colluricincla megarhyncha*. Maximum likelihood tree from mitochondria DNA showing estimated phylogenetic relationships among putative geographic basins and smaller islands surrounding the Island of New Guinea. Numbers above branches indicate Bayesian posterior probabilities greater than 0.95, number below branches are bootstrap values greater than 75%.

The maximum likelihood tree and Bayesian analysis of the mtDNA support five distinct clades on mainland New Guinea and three well supported lineages on surrounding islands (Figure 4). For discussion purposes, we refer to these clades using the name of the largest basin in which they are found, and these are shown in Figure 4. We found no disagreement between the well-supported nodes in the MrBAYES analysis (Bayesian posterior probability of 0.95 or greater) and the well-supported nodes from the maximum likelihood bootstrap values (bootstrap values of 75% or greater). The likelihood tree and Bayesian tree topology matched our hypothesized tree (Figure 2, number below refer to those in figure) with the following two exceptions; first, the Bintuni Basin (1) clade contains haplotypes from the South Papuan Basin (6) clade (Figure 4), whereas we hypothesized that BB would be sister to clades found in the north (MB (2) and SRB (3)) through the dispersal route formed from the filling in of Aetna Bay (-ab). Second, the Cape Vogel Basin (4) contains haplotypes from the South Papuan Peninsula (5) clade (Figure 4), rather than forming a separate sister lineage.

### Phylogeographic network relationships for mtDNA and nDNA

**Figure 5:**
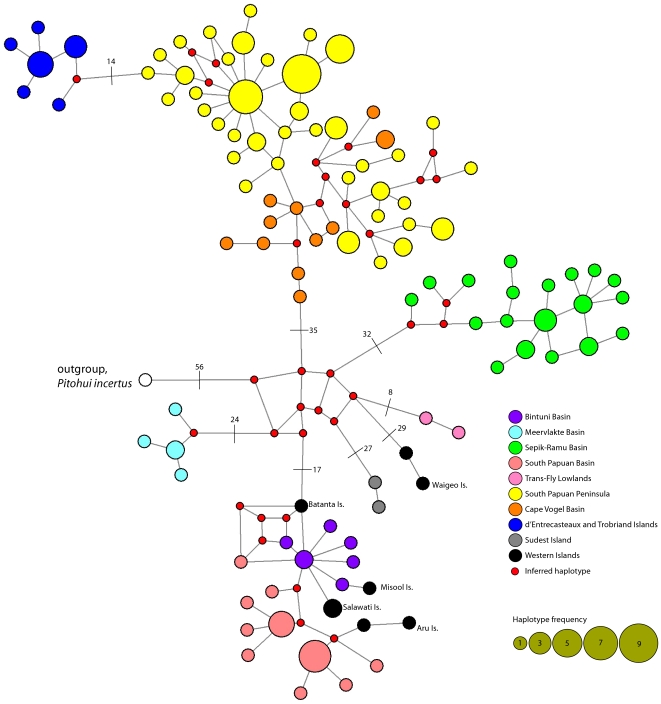
Relationships of mtDNA haplotypes among samples collected throughout New Guinea and surrounding islands. Network of haplotypes from mtDNA (concatenated CytB, ND2 and ATPase 8; 572 bp). Slash marks across lines and adjacent numbers indicate the number of base pair changes between the two haplotypes; all other lines represent one base pair change. White haplotype is the outgroup *P. incertus*.

**Figure 6:**
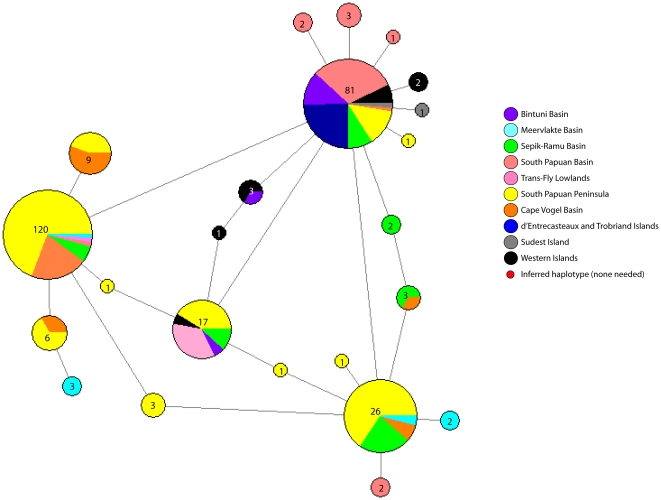
Relationships of nDNA alleles among samples collected throughout New Guinea and surrounding islands. Network of nuclear alleles from AR35 (allele length is 138 bp). Number in pie chart indicates the number of alleles and all lines indicate one base pair change.

The mtDNA haplotype network illustrates eight major haplotype groups separated by at least 8 base pairs and some as great as 35 base pairs between basins (Figure 5). The nuclear allele network showed some structure among geographic basins (Figure 6) even though basins are not monophyletic. Additionally, the allele network illustrates significant genetic variation at this locus with 23 unique alleles in a 138 bp region (see Figure S1 for estimate of congruence between mtDNA and nuclear locus). Furthermore, this is an underestimate of diversity given that 46 alleles from heterozygotes could not be phased.

### Molecular variance for mtDNA and nDNA

The mtDNA AMOVA found significant support for the greatest variance being explained when partitioned among the seven lowland basins (Table 1 lists basins; among basins 95.8% of the variation was explained, Φ_ct_ = 0.96, p<0.05; 1.8% of the variation explained among sites within basins, Φ_st_ = 0.98, p<0.05; 2.4% variation explained within sites Φ_sc_ = 0.43, p<0.05). The pairwise analysis of F_st_ also supports a high degree of genetic differentiation for both mtDNA and nDNA among lowland basins ranging from 0.30–0.96 and 0.10–0.52 respectively and excluding non-significant values (Table 3a and b). All pairwise F_st_ values were significant except TF to MB for mtDNA and BB to SPB and CVB to SPP for nDNA. TF and MB non-significance was likely due to low sample numbers for these basins (see Table S1). Differentiation between the CVB and the SPP was the lowest at 0.30 for mtDNA and −0.01 (not significant) for nDNA, whereas the majority of F_st_ estimates were above 0.80 for mtDNA and 0.20 for nDNA (Table 3a and b).

**Table 3:** Pairwise F_st_ among basins.

A) mtDNA							
	SPP	SPB	BB	MB	TF	SRB	CVB
SPP	0						
SPB	0.89	0					
BB	0.88	0.55	0				
MB	0.81	0.89	0.95	0			
TF	0.88	0.90	0.96	0.95*	0		
SRB	0.89	0.93	0.94	0.88	0.93	0	
CVB	0.30	0.93	0.95	0.90	0.94	0.94	0

B) nuclear DNA AR53							
	SPP	SPB	BB	MB	TF	SRB	CVB
SPP	0.00						
SPB	0.34	0.00					
BB	0.36	0.00*	0.00				
MB	0.33	0.42	0.39	0.00			
TF	0.36	0.30	0.28	0.32	0.00		
SRB	0.17	0.10	0.11	0.20	0.18	0.00	
CVB	−0.01*	0.46	0.52	0.31	0.46	0.21	0.00

*not significant at the p<0.05 level, Abbreviations for basins in Table S1.

### Divergence dating for mtDNA only

Due to small sequence length obtained (138 bp) for the nuclear dataset (modern and historic samples), divergence dating was only performed with mtDNA data. Estimates of K2P genetic distances among basins were relatively high and significant. Corrected pairwise genetic distances ranged from 1% to 11% (Table 4). With the exception of sites in MB, molecular divergence estimates among birds sampled from sites on either side of the Central Ranges span the estimated timing of their uplift of 4–5 Mya (Table 1 and 4). SRB is highly divergent from basins south of the central ranges (9–10%, 1.7–5.1 Mya, Table 4). CVB and SPP had low pairwise divergence (1%, 0.3–1.0 Mya, Table 4) and so did BB and SPB (1%, 0.4–0.7 Mya, Table 4). The TF lowlands are similarly divergent from the western and southern basins (6% Table 4, BB and SPB respectively) and an estimate split time around the early Pleistocene (1.7–4.4 Mya); however, they are highly divergent from the southeastern and northern basins (except MB) with an estimated divergence of 9–10%. The TF haplotypes were not significantly differentiated from the sample from Cape York, Australia (genetic distance of 0.001; not in table but see branch length between Cape York and Wuroi-Oriomo River samples in Figure 4).

**Table 4:** Genetic distance between basins and estimated age of mtDNA lineage.

Basin	SPP	SPB	BB	MB	TF	SRB	CVB
SPP	**0.7**	0.10	0.10	0.06	0.10	0.10	0.01
SPB	2.1, 4.8	**0.3**	0.01	0.05	0.06	0.09	0.10
BB	2.1, 4.8	0.7, 0.4	**0.1**	0.06	0.06	0.09	0.10
MB	2.1, 2.9	1.7, 2.6	1.7, 2.7	**0.2**	0.05	0.05	0.06
TF	2.1, 4.9	1.4, 3.0	1.7, 2.7	1.7, 2.4	**0.1**	0.09	0.10
SRB	2.1, 4.7	1.7, 4.3	1.4, 4.5	1.4, 2.5	1.7, 4.4	**0.3**	0.11
CVB	1.0, 0.3	2.1, 4.9	2.1, 4.9	2.1, 3.0	2.1, 4.9	2.1, 5.1	**0.4**

Corrected pairwise K2P genetic distance among basins (above diagonal), pairwise genetic distance within basins (diagonal using single clock estimate) and molecular clock estimates from BEAST and an assumed molecular clock of 2.1% per Mya respectively (below diagonal).

### Isolation by distance and population expansion with mtDNA only

**Figure 7:**
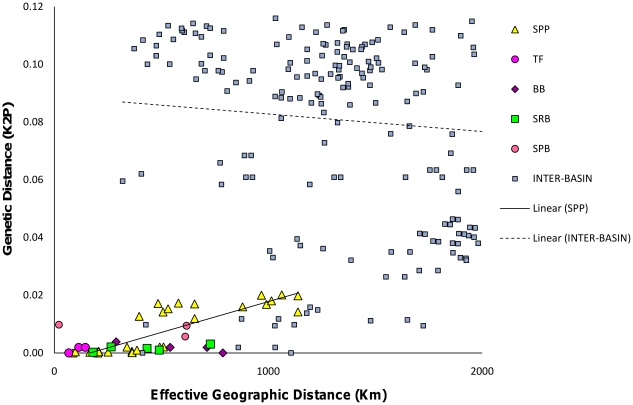
Estimates of genetic isolation correlated with their geographic distance. Isolation by distance estimates for South Papuan Peninsula (SPP, yellow), Transfly Basin (TF, dark pink), Bintuni Basin (BB, purple), Sepik-Ramu (SRB, green); South Papuan Basin (SPB, pink). Cape Vogel (CVB) and Meervlakte (MB) are not depicted since they only have two sites in each basin. Blue squares are comparisons between basins. The Mantel test was significant at p<0.05, but slopes indicate positive correlation within basins (solid line fitted to SPP only) and a negative correlation between basins (dashed line fitted to all inter-basin comparisons).

Correlation tests revealed positive significant patterns of isolation by distance across all sites (Mantel test p<0.05, Figure 7). However, the correlation coefficient was 0.40 and the amount of genetic differentiation explained by geographic distance is only 16%. When intra- and inter-basins are separated, some within basin patterns show a positive slope and between basins show a negative slope (SPP in Figure 7). Nucleotide diversity (π) is lowest within BB and TF, moderate within SPB, CVB, and MB and highest within SRB and SPP (Table S4). Tajima's D estimates were non-significant and negative, except the two with the lowest sample sizes (Table S4). Fu's F_s_ values were not significant except for BB (Table S4).

### Evolution of Dispersal estimated with mtDNA only

**Figure 8:**
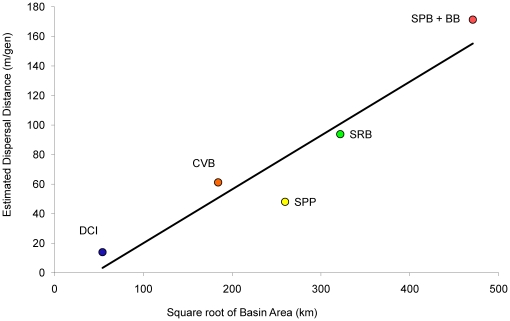
Correlation of range size and estimated dispersal distance for genetic lineages in *Colluricincla megarhyncha*. Represents the estimated dispersal distance for each clade correlated with the area of the putative range within basins in New Guinea and an island group d'Entrecasteaux Islands. Location abbreviations are in Table S1 (except DCI: d'Entrecasteaux Islands), colors match all other figures. Correlation is among basin/island size and dispersal distance estimates from Phylomapper v1beta1 (Lemmon and Lemmon 2008). R = 0.948, p = 0.014. Line indicates positive relationship only and is not meant to infer causation in the form of a regression.

We found strong evidence for evolution in dispersal distance with statistical support for the most complex model, which contained six dispersal classes (Table S3). The per-generation dispersal distance was highest for the SPB and BB clade and lowest for the d'Entrecasteaux Islands clade. We also found a significant positive correlation between dispersal distance and estimated range size (Figure 8, *R* = 0.948, *p* = 0.014).

## Discussion

Our study reveals a remarkable level of genetic divergence within *C. megarhyncha*. The high level of divergence (5–11% from combined mtDNA loci) observed among *C. megarhyncha* lineages are similar to the levels separating different genera and even families of birds in North America [53], [54]. Furthermore, the congruence between the mtDNA and nuclear intron data indicates that lineages have been isolated long enough for sorting to occur given the expected difference in coalescent time between the two genomes. A recent review of congruence between genomes by Zink and Barrowclough [55] shows nicely the patterns expected between markers and whether enough time has passed for lineage sorting to occur, see Box 2 and Figure 1 in citation [55]. We have compared our data using their framework, and we conclude that the genes sampled here are congruent (Figure S1).

Thirty subspecies have been described within *C. megarhyncha*, but the genealogical exclusivity of these subspecies, or their interrelationships, had not been examined with genetic data. Data from this study of 23 of those subspecies demonstrates there are eight highly divergent lineages that are geographically distributed in regions corresponding to historically isolated lowland basins or islands, and these do not always correspond to currently recognized subspecies patterns. Furthermore, levels of genetic divergence estimated in this study (27.96–59.77; corrected K2P genetic distances) among the eight lineages are much higher than those found among congeneric species (0.78–11.77 corrected K2P genetic distance, see discussion in Tavares and Baker [56] and Hebert et al. [53]). The amount of divergence among lineages with *C. megarhyncha* warrants further study to consider elevating any to full species. Such a study should include subspecies we did not sample, comparisons of individual life history characteristics (e.g., song and behavior) and pre- and post-zygotic isolating mechanisms to determine any additional ecological and genetic characteristics that are important in isolating the eight putative lineages found in this study.

### *C. megarhyncha's* evolution and New Guinea's geologic history

The mountain-forming events in New Guinea correspond with our molecular clock estimates of time since divergence, F_st_ estimates, and phylogenetic structure (geologic dates in Table 1, molecular estimates in Table 4). Information regarding orogeny was derived from independent sources [16], [17], [18]; however, synonymizing molecular clock estimates with geologic estimates remains contentious. The controversy stems from an assumption that a single mutation rate among all sites and across multiple mtDNA genes in birds may or may not always be valid most recently discussed in [57], [58]. The largest concerns are whether the genes evolve in a clock-like fashion and whether the correct rate is assumed [57]. A recent review, however, of 90 candidate clocks that have been proposed for birds, found that a mutation rate of 2.1% (±0.1%) for cytochrome *b* is a stable estimate for mutations occurring in the last 12 Mya [47]. We used this robust molecular clock estimate as well as estimates derived from a Bayesian model for rate variation among lineages. While the BEAST estimates are all lower, the range estimated between the two methods falls within the range estimated for timing from the geologic literature, which itself has error in estimated dates. Because the ages of multiple mtDNA lineages roughly coincide with age estimates for several relevant barriers (except those between BB and SPB and those between SPP and CVB), we believe that vicariance barriers helped to isolate multiple *Colluricincla* lineages.

The best supported vicariance barrier is the Central Ranges, which results in a phylogenetic division between sites to the north and south of these mountains. The Central Range uplift is estimated to have begun around 4–5 Mya [17], [18], [59]. The range of dates estimated for molecular divergence among clades on either side of the Central Ranges indicates that lineages have been isolated since the early Pliocene.

This vicariant pattern across the Central Ranges is concordant with other taxa on New Guinea, including rainbowfish, family Melanotaeniidae [60], Owlet-nightjars, family Aegothelidae [30], and bowerbirds, family Ptilonorhynchidae [61], which have high molecular divergence estimates and are similar to those estimated here. Palm Cockatoos, *Probosciger aterrimus*, however, do not show strong differentiation across the Central Ranges suggesting that some species may have arrived and spread across the island after the Central Ranges formed [62]. Although there are only a few published papers, these independent tests across multiple lineages with genetic data from birds and fish species support the hypothesis that the Central Ranges create a vicariant barrier for many taxa with lowland distributions on New Guinea.

Another notable vicariance pattern was that observed among the South Papuan Basin and the Trans-Fly Lowlands south of the Central Ranges. In the southern lowlands, ecological divergence in climate and vegetation may have caused a decrease in gene flow for many populations [63], [64], [65]. During the past two million years, sea levels fluctuated such that Australia and New Guinea were either connected or isolated by seas, with the most recent connection occurring about 21,000 years ago [66]. Molecular divergence estimates between SPB and TF suggest that these basins diverged between 1.4–3.0 Mya, supporting the role of fluctuating sea levels as a barrier to gene flow. Additionally, there is no significant divergence among the TF lowlands and Cape York (*C. megarhyncha* from the northern tip of Australia), which implies that they diverged recently, there is substantial gene flow from Australia, or the Australian form is a recent migrant from New Guinea.

As an alternative to the geographic vicariance model, ecological vicariance may also affect this area between South Papuan Basin and the Trans-Fly Lowlands. Lowlands in this region include dry savanna-like habitats that are unsuitable for *C. megarhyncha*. This unsuitable habitat may act as an ecological barrier to dispersal, and further restrict gene flow between the SPB and TF. There are no publications containing data on the timing or historical extent of these savanna-like habitats, so at this time we are not able to test the timing of ecological changes with genetic data.

Lastly, the Aure Trough was an inland sea that filled around 3–4 Mya, however the dates are disputed [13], [14], [15]. Genetic evidence here suggest a divergence time of 2.1–4.9 Mya between the SPP and SPB providing evidence that the Aure Trough was a barrier to dispersal in the past. However, the SPP is hypothesized to have formed a land bridge between Australia and the mainland of New Guinea around 5 Mya, which also coincides with evidence from molecular data in this study. Therefore, it is unclear whether the SPP diverged on an island that became part of mainland New Guinea or whether the Aure Trough separated once connected populations that diverged in allopatry. Regardless, there is a strong clear break between lineages from sites in the SPB and SPP which today are in continuous rainforest habitat.

Nuclear data, while not as strongly structured as mtDNA, still support many of the observed patterns in the mtDNA dataset. Systematic biases in estimates of diversity were created by removing unresolved nuclear alleles from the dataset [67]. Therefore, the values reported here should be treated with caution. This bias, however, is conservative because estimates of F_st_ and gene diversity would be higher had we been able to phase all alleles sampled.

### Support for founder effect with dispersal among basins

There are two basins with phylogenetic signatures that are similar to that expected under the founder effect with dispersal across New Guinea. These are (1) CVB and SPP and (2) SPB and BB. First, the topology of the phylogenetic tree indicates that samples from CVB are found within the larger SPP clade. This is a typical pattern observed when a small portion of the haplotype diversity moves into a new area through dispersal, see Figure 1C in [4] and is also a pattern that has been found in several suspected island-hopping taxa [4], [45], [68], [69], [70]. Northwestern movement of birds in CVB is hindered by the Huon Peninsula (estimated uplift occurred 1.5 Mya) and molecular clock estimates between CVB and SRB are too high (2.1–5.1 Mya) in order for this to have been a vicariant barrier separating them. Therefore, the FED hypothesis is more likely and birds from CVB sites were probably founded by birds from SPP (given the low divergence between these two basins) and the Central Ranges account for the difference between CVB and SRB. Lastly, the F_st_ pairwise estimates from both the mtDNA and nDNA between CVB and SPP indicate much lower differentiation, and in the case of nDNA are non-significant and F_st_ is zero. It should be noted, however, that the number of sites sampled within the CVB was low (only two) and further sampling could reveal a different pattern.

The second group reflecting a FED pattern is SPB and BB basins. The haplotypes sampled from the eastern side (Ekame) of the SPB are found within the diversity of haplotypes sampled within SPB and BB. This node is not significant in the Bayesian analysis (0.82); therefore, support for the FED hypothesis in this geographic region is weak compared to that of SPP and CVB. Molecular clock estimates between SPB and BB are low (0.4–0.7 Mya) and F_st_ values are low or non-significant for both mtDNA and nDNA across the western expanse of the island indicating that there are not many historical barriers to gene flow. Lastly, the Fu F_s_ test of recent expansion was significant for only BB, but both D and F_s_ are negative indicating a possible recent expansion.

### Support for isolation by distance within basins

Within each major clade, correspondence to geographic basins was evident and birds sampled from multiple sites within a basin had lower molecular diversity and less population genetic structure within compared to among basins (except those showing founder effects; CVB and SPB). Isolation by distance analysis was significant, but when the intra- versus inter-basin comparison is separated, it becomes apparent that the within basin comparisons are likely driving the significant pattern of isolation by distance. In Figure 7, the intra-basin comparison for SPP shows a positive correlation with geographic distance, but when sites are compared between basins the slope is negative. This indicates that gene flow between basins is unlikely. Therefore, IBD is likely the weakest mechanism that has driven diversification across most basins on the island, but is potentially a mechanism that generates variation among sites within basins. The current number of sites sampled within most of the basins is low, however, and would need to be increased for better statistical power to detect isolation by distance within most basins.

### Conclusions

Like many lowland Papuan species, *Colluricincla megarhyncha* shows tremendous subspecific variation. Their current classification as polytypic species reflects the complex and confusing patterns of variation in the region; first, the assumption that populations are connected in the lowlands, and second, the historical preference of avian taxonomists to use polytypic species concepts and trinomials in classification. For example, perhaps the most influential compilation of avian taxonomic names in New Guinea was Ernst Mayr's checklist that includes 22 races of *C. megarhyncha* [71]. Because of relatively poor geographic sampling and attention to this region, more-recent treatments have been unable to improve upon these early classifications, and current work continues to utilize polytypic species and trinomials in the region [24], [72]. Here we clearly show that genetic data are powerful not only for identifying distantly related groups (that should likely be elevated to full species), but also for helping to identify the evolutionary processes responsible for their diversification such as the evolution in dispersal distance between clades.

For ornithologists and conservationists, it is important to recognize that the current taxonomy of polytypic species should often be considered a place-holder and working hypothesis until more data can be leveraged to test biological and phylogenetic species boundaries. Many polytypic species are likely “oversplit” [73], but it is often impossible to know which splits are significant without additional geographical sampling and additional testing of molecular, behavioral, ecological, etc., traits, for example see [74]. Our data support this assertion and offer data that can be used for taxonomic solutions for *C. megarhyncha*. Our work additionally warns that even lowland taxa with continuous ranges are subject to major taxonomic revision. In many cases, currently-recognized species probably include multiple good biological species and/or phylogenetic species, but additional work will be required to reliably identify functional biological units.

### Conservation Implications

The data presented here support several potential isolating barriers to gene flow on the island of New Guinea. First, our study has helped identify phylogeographic basins within lowland rainforests. This information is important for identifying and prioritizing conservation areas and is currently underutilized in conservation planning [75], [76]. Furthermore, conservation biologists recognize the importance of conserving “evolutionary potential” and evolutionarily significant units [74], [76]: thus, conservation planning requires data about species in a phylogenetic and phylogeographic context to make more informed decisions about species conservation. Our study is among the first to do so for a lowland species complex for the island of New Guinea and satellite islands.

## Supporting Information

Figure S1 **Consistent divergence results between nuclear and mitochondrial DNA.** F_st_ data from Table 3 graphed following that of Zink and Barrowclough [55] to illustrate congruence between mtDNA and nuclear estimates of divergence. From Zink and Barrowclough [55] “categories A and D indicate generally consistent results between mitochondrial and nuclear markers, category B results are consistent given differences in effective population size and coalescent times, and category C results are inconsistent.” The two outlier points in the “B” section are those comparisons between SPP and CVB and SPB and BB which show evidence of isolation by distance or founder effect with dispersal from other analysis (see such sections in text).
(TIF)

Table S1 **Location of sites sampled in New Guinea and surrounding islands.**
(XLS)

Table S2 **Detailed specimen data for all samples used in this study.**
(XLS)

Table S3 **Phylomapper estimates of evolution in dispersal distance among basins.**
(XLS)

Table S4 **Molecular diversity estimates and tests of recent population expansion.**
(XLS)

## References

[1] Avise JC (2000). Phylogeography: the history and formation of species.

[2] Cracraft J (1983). Species concepts and speciation analysis.. Current Ornithology.

[3] Cracraft J (1986). The origin and early diversification of birds.. Paleobiology.

[4] Wagner WL, Funk VA (1995). Hawaiian biogeography. Evolution on a hot spot archipelago.

[5] Zink RM, Remsen JV (1986). Evolutionary processes and patterns of geographic variation in birds.. Current Ornithology.

[6] Zink RM (1996). Comparative phylogeography in North American birds.. Evolution.

[7] Wallace A (1869). The Malay Archipelago.

[8] Mayr E (1942). Systematics and the Origin of Species.

[9] Mayr E (1963). Animal species and evolution.

[10] Mack A, Dumbacher J (2007). Birds of Papua.. The Ecology of Papua Part.

[11] Beehler BM, Pratt TK, Zimmerman DA (1986). Birds of New Guinea: Princeton University Press..

[12] Slater P (1986). The Slater field guide to Australian birds: Rigby, Sydney.

[13] Abbott L, Silver E, Anderson R, Smith R, Ingle J (1997). Measurement of tectonic surface uplift rate in a young collisional mountain belt.. Nature.

[14] Davies JM, Dunne RP, Brown BE (1997). Coral bleaching and elevated sea-water temperature in Milne Bay Province, Papua New Guinea, 1996.. Marine and Freshwater Research.

[15] Dow DB (1977). A geological synthesis of Papua New Guinea. Bureau of Mineral Resources Bulletin.

[16] Dow DB, Sukamto R (1984). Western Irian Jaya: the end-product of oblique plate convergence in the late Tertiary.. Tectonophysics.

[17] Pigram CJ, Davies HL (1987). Terranes and the accretion history of the New Guinea orogen.. BMR Journal of Australian Geology and Geophysics.

[18] Pigram CJ, Symonds PA (1991). A review of the timing of the major tectonic events in the New Guinea Orogen.. Journal of Southeast Asian Earth Sciences.

[19] Stevens C, McCaffrey R, Silver EA, Sombo Z, English P (1998). Mid-crustal detachment and ramp faulting in the Markham Valley, Papua New Guinea.. Geology.

[20] Greenway JC, Mayr E, Moreau RE, Rand AL, Salomonsen F (1967). Check-list of Birds of the World: a continuation of the work of James L. Peters.

[21] Ford J (1979). Subspeciation, hybridization and relationships in the Little Shrike-thrush *Colluricincla megarhyncha* of Australia and New Guinea.. Emu.

[22] Bell HL (1982a). A bird community of lowland rainforest in New Guinea. 2. Seasonality.. Emu.

[23] Bell HL (1982b). A bird community of lowland rainforest in New Guinea. I. Composition and density of the avifauna.. Emu.

[24] Higgins PJ, Peter JM (2002). Handbook of Australian, New Zealand & Antarctic Birds. Vol. 6, Pardalotes to Shrike-thrushes.

[25] Mack AL, Wright D (1996). Notes on occurrence and feeding of birds at Crater Mountain Biological Research Station, Papua New Guinea.. Emu.

[26] Dumbacher JP, Deiner K, Thompson L, Fleischer RC (2008). Phylogeny of the avian genus Pitohui and the evolution of toxicity in birds.. Molecular Phylogenetics and Evolution.

[27] Voris HK (2000). Special paper 2: maps of Pleistocene sea levels in Southeast Asia: Shorelines, river systems and time durations.. Journal of Biogeography.

[28] Greenberg R, Cordero PJ, Droege S, Fleischer RC (1998). Morphological adaptation with no mitochondrial DNA differentiation in the coastal plain swamp sparrow.. Auk.

[29] Sorenson MD, Cooper A, Paxinos EE, Quinn TW, James HF (1999). Relationships of the extinct moa-nalos, flightless Hawaiian waterfowl, based on ancient DNA.. Proceedings of the Royal Society B: Biological Sciences.

[30] Dumbacher JP, Pratt TK, Fleischer RC (2003). Phylogeny of the owlet-nightjars (Aves: Aegothelidae) based on mitochondrial DNA sequence.. Molecular Phylogenetics and Evolution.

[31] Kocher TD, Thomas WK, Meyer A, Edwards SV, Pääbo S (1989). Dynamics of mitochondrial DNA evolution in animals: amplification and sequencing with conserved primers.. Proceedings of the National Academy of Sciences.

[32] Stephens M, Smith NJ, Donnelly P (2001). A new statistical method for haplotype reconstruction from population data.. The American Journal of Human Genetics.

[33] Posada D (2008). jModelTest: phylogenetic model averaging.. Molecular Biology and Evolution.

[34] Guindon S, Gascuel O (2003). A simple, fast, and accurate algorithm to estimate large phylogenies by maximum likelihood.. Systematic Biology.

[35] Zwickl DJ (2006). Genetic algorithm approaches for the phylogenetic analysis of large biological sequence datasets under the maximum likelihood criterion.. The University of Texas at Austin.

[36] Nielsen R, Huelsenbeck JP, Ronquist F (2005). Bayesian Analysis of Molecular Evolution Using MrBayes.. Statistical Methods in Molecular Evolution.

[37] Excoffier L, Laval G, Schneider S (2007). Arlequin ver. 3.11.

[38] Bandelt HJ, Yao YG, Bravi CM, Salas A, Kivisild T (2009). Median network analysis of defectively sequenced entire mitochondrial genomes from early and contemporary disease studies.. Journal of Human Genetics.

[39] Drummond A, Ho S, Phillips M, Rambaut A (2006). Relaxed phylogenetics and dating with confidence.. PLoS Biology.

[40] Drummond A, Rambaut A (2007). BEAST: Bayesian evolutionary analysis by sampling trees.. BMC Evolutionary Biology.

[41] Kimura M (1980). A simple method for estimating evolutionary rates of base substitutions through comparative studies of nucleotide sequences.. Journal of Molecular Evolution.

[42] Jin L, Nei M (1990). Limitations of the evolutionary parsimony method of phylogenetic analysis.. Molecular Biology and Evolution.

[43] Hillis DM, Moritz C, Mable BK, Olmstead RG (1996). Molecular systematics: Sinauer Sunderland, MA.

[44] Nei M, Jin L (1989). Variances of the average numbers of nucleotide substitutions within and between populations.. Molecular Biology and Evolution.

[45] Edwards SV (1997). Relevance of microevolutionary processes to higher level molecular systematics.

[46] Fleischer RC, McIntosh CE, Tarr CL (1998). Evolution on a volcanic conveyor belt: using phylogeographic reconstructions and K-Ar-based ages of the Hawaiian Islands to estimate molecular evolutionary rates.. Molecular Ecology.

[47] Weir JT, Schluter D (2008). Calibrating the avian molecular clock.. Molecular Ecology.

[48] Fu Y (1997). Statistical tests of neutrality of mutations against population growth, hitchhiking and background selection.. Genetics.

[49] Tajima F (1989). The effect of change in population size on DNA polymorphism.. Genetics.

[50] Ray N (2005). PATHMATRIX: a geographical information system tool to compute effective distances among samples.. Molecular Ecology Notes.

[51] Lemmon AR, Lemmon EM (2008). A likelihood framework for estimating phylogeographic history on a continuous landscape.. Systematic Biology.

[52] Sanderson M (1997). A nonparametric approach to estimating divergence times in the absence of rate constancy.. Molecular Biology and Evolution.

[53] Hebert P, Stoeckle M, Zemlak T, Francis C (2004). Identification of birds through DNA barcodes.. PLoS Biology.

[54] Kerr KCR, Stoeckle MY, Dove CJ, Weigt LA, Francis CM, Hebert PDN (2007). Comprehensive DNA barcode coverage of North American birds.. Molecular Ecology Notes.

[55] Zink RM, Barrowclough G (2008). Mitochondrial DNA under siege in avian phylogeography.. Molecular Ecology.

[56] Tavares E, Baker A (2008). Single mitochondrial gene barcodes reliably identify sister-species in diverse clades of birds.. BMC Evolutionary Biology.

[57] Lovette IJ (2004). Mitochondrial dating and mixed support for the 2% rule in birds.. Auk.

[58] Peterson AT (2006). Application of molecular clocks in ornithology revisited.. Journal of Avian Biology.

[59] Heads M (2002). Birds of paradise, vicariance biogeography and terrane tectonics in New Guinea.. Journal of Biogeography.

[60] McGuigan K, Zhu D, Allen G, Moritz C (2000). Phylogenetic relationships and historical biogeography of melanotaeniid fishes in Australia and New Guinea.. Marine and Freshwater Research.

[61] Zwiers PB, Borgia G, Fleischer RC (2008). Plumage based classification of the bowerbird genus Sericulus evaluated using a multi-gene, multi-genome analysis.. Molecular Phylogenetics and Evolution.

[62] Murphy SA, Double MC, Legge SM (2007). The phylogeography of palm cockatoos, Probosciger aterrimus, in the dynamic Australo-Papuan region.. Journal of Biogeography.

[63] Hope G, Keast A, Miller SE (1996). Quaternary change and the historical biogeography of Pacific Islands.. The origin and evolution of Pacific island biotas, New Guinea to Eastern Polynesia: patterns and processes.

[64] Hope G, Tulip J (1994). A long vegetation history from lowland Irian Jaya, Indonesia.. Palaeogeography, Palaeoclimatology, Palaeoecology.

[65] Kershaw AP (1994). Pleistocene vegetation of the humid tropics of northeastern Queensland, Australia.. Palaeogeography, Palaeoclimatology, Palaeoecology.

[66] Jennings JN, Mulvaney DJ, Golson J (1971). Sea level changes and land links.. Aboriginal Man and Environment in Australia.

[67] Garrick RC, Sunnucks P, Dyer RJ (2010). Nuclear gene phylogeography using PHASE: dealing with unresolved genotypes, lost alleles, and systematic bias in parameter estimation.. BMC Evolutionary Biology.

[68] Baldwin BG, Robichaux RH, Wagner W, Funk V (1995). Historical biogeography and ecology of the Hawaiian silversword alliance (Asteraceae): new molecular phylogenetic perspectives.. Hawaiian biogeography: Evolution in a Hotspot Archipelago.

[69] Shaw KL, Wagner W, Funk V (1995). Biogeographic patterns of two independent Hawaiian cricket radiations (*Laupala* and *Prognathogryllus*).. Hawaiian Biogeography.

[70] Tarr CL, Fleischer RC, Wagner W, Funk V (1995). Evolutionary relationships of the Hawaiian honeycreepers (Aves, Drepanidinae).. Hawaiian Biogeography.

[71] Mayr E (1941). List of New Guinea Birds: a systematic and faunal list of the birds of New Guinea and adjacent islands.

[72] Mayr E, Diamond J (2001). The birds of northern Melanesia: speciation, ecology & biogeography.

[73] Zink RM (2004). The role of subspecies in obscuring avian biological diversity and misleading conservation policy.. Proceedings of the Royal Society B: Biological Sciences.

[74] Crandall K, Bininda-Emonds O, Mace G, Wayne R (2000). Considering evolutionary processes in conservation biology.. Trends in Ecology and Evolution.

[75] de Guia APO, Saitoh T (2007). The gap between the concept and definitions in the evolutionarily significant unit: the need to integrate neutral genetic variation and adaptive variation.. Ecological Research.

[76] Moritz C, Richardson K, Ferrier S, Monteith G, Stanisic J (2001). Biogeographical concordance and efficiency of taxon indicators for establishing conservation priority in a tropical rainforest biota.. Proceedings of the Royal Society of London Series B: Biological Sciences.

