# Supplementary figures and images for: A Passerine Bird's Evolution Corroborates the Geologic History of the Island of New Guinea
Source: PLoS One. 2011 May 6;6(5):e19479. doi: 10.1371/journal.pone.0019479 (PMC3089620; doi:10.1371/journal.pone.0019479)

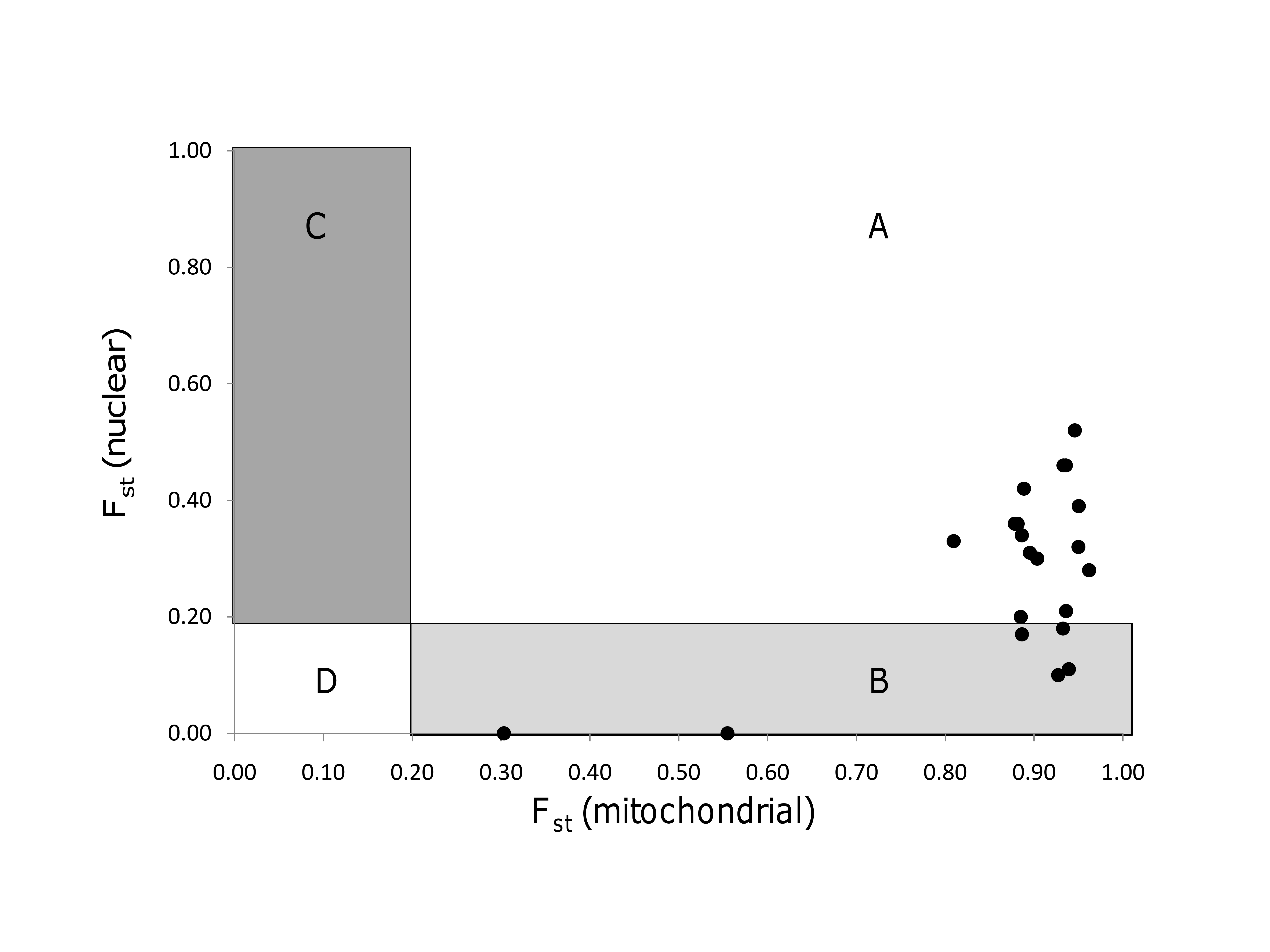

Supplement: Figure S1 — Consistent divergence results between nuclear and mitochondrial DNA. Fst data from Table 3 graphed following that of Zink and Barrowclough [55] to illustrate congruence between mtDNA and nuclear estimates of divergence. From Zink and Barrowclough [55] “categories A and D indicate generally consistent results between mitochondrial and nuclear markers, category B results are consistent given differences in effective population size and coalescent times, and category C results are inconsistent.” The two outlier points in the “B” section are those comparisons between SPP and CVB and SPB and BB which show evidence of isolation by distance or founder effect with dispersal from other analysis (see such sections in text). (TIF) [file pone.0019479.s001.tif]
